# Combination of Periodontal Ligament Stem Cells and Metformin via Organic Cation Transporters for Periodontal Regeneration in Rats

**DOI:** 10.3390/biom15050663

**Published:** 2025-05-03

**Authors:** Qingchen Qiao, Zeqing Zhao, Yaxi Sun, Jing Wang, Xiaowei Li, Li Zhang, Hao Yang, Ning Zhang, Ke Zhang, Yuxing Bai

**Affiliations:** Department of Orthodontics, School of Stomatology, Capital Medical University, Beijing 100070, China; morning@mail.ccmu.edu.cn (Q.Q.); zqzhao@mail.ccmu.edu.cn (Z.Z.); sunyaxi@mail.ccmu.edu.cn (Y.S.); kwwjjxa@mail.ccmu.edu.cn (J.W.); xiaoweibernice@163.com (X.L.); zhangli_cpums@163.com (L.Z.); sy_yangh@mail.ccmu.edu.cn (H.Y.); dentistzhang112@ccmu.edu.cn (N.Z.)

**Keywords:** organic cation transporters, human periodontal ligament stem cells, metformin, osteogenesis, cementogenesis, periodontal regeneration, rat model

## Abstract

Periodontal regeneration remains challenging due to individual variability, especially in treatments involving bioactive factors such as metformin. This study aimed to investigate the role of organic cation transporters (OCTs) in metformin-induced periodontal regeneration. The expression and function of OCTs in human periodontal ligament stem cells (hPDLSCs) were assessed, and OCT-mediated metformin uptake was quantified by high-performance liquid chromatography (HPLC). Osteogenic and cementogenic differentiation markers were analyzed in vitro, and periodontal regeneration was evaluated using a rat periodontal defect model. OCTs were differentially expressed and functional in hPDLSCs. Both the OCT1 inhibitor cimetidine and OCT1 knockdown significantly reduced intracellular metformin accumulation to 50–60% and 20–30% of control levels, respectively (*p* < 0.01). Cimetidine diminished the osteogenic and cementogenic effects of metformin by approximately 31–48% and 32–40%, respectively (*p* < 0.01). In vivo, oral administration of cimetidine decreased bone regeneration by 25% and cementum regeneration by 36% compared with controls receiving GelMA/hPDLSCs/metformin (*p* < 0.01). This study demonstrates that OCTs regulate metformin uptake in hPDLSCs, and that inhibition of OCT1 by cimetidine significantly reduces the osteogenic and cementogenic efficacy of metformin, providing the first evidence of drug interactions affecting periodontal regeneration mediated by OCT transport in rats.

## 1. Introduction

Metformin, a widely prescribed biguanide, is recognized as the first-line treatment for type 2 diabetes mellitus in international clinical guidelines [1,2]. In addition to its established antidiabetic effects, metformin exhibits therapeutic potential in various metabolic disorders, including anti-aging, anticancer, and cardioprotective properties [3,4]. Recent studies have revealed the capacity of metformin to promote periodontal regeneration [5,6]. Periodontal defects, characterized by damage to the periodontal complex (including cementum, periodontal ligament, and alveolar bone), have been reported to be the sixth-most prevalent condition worldwide [7]. However, variability in individual responses highlights the need to clarify metformin’s mechanisms to enhance its regenerative effects and clinical utility.

Periodontal ligament stem cells (PDLSCs) are among the most promising candidates for periodontal tissue repair in tissue engineering [8,9,10]. hPDLSCs can be readily obtained from extracted wisdom teeth, supernumerary teeth, or orthodontic extractions. This low-cost and minimally invasive process makes them highly accessible [11,12]. Previous research reported that hPDLSCs from the same vial have the capability to differentiate into osteoblasts, fibroblasts, and cementoblasts, which demonstrated their potential to form the bone–PDL–cementum complex [9].

In our previous research, we developed a novel injectable and load-bearing CPC–hPDLSC–fibers–metformin construct for dental and craniofacial bone tissue engineering. CPC + hPDLSCs + 0.1%Met group demonstrated enhanced osteogenic differentiation of hPDLSCs [13]. Our findings also indicate that metformin, at an optimal concentration of 100 μM, enhances the osteogenic and cementogenic differentiation of hPDLSCs. Furthermore, combining metformin with hPDLSCs effectively promotes the regeneration of both alveolar bone and cementum, resulting in the restoration of functional periodontium.

However, clinical observations indicate that only approximately 60% of T2DM patients respond effectively to metformin, with reduced efficacy potentially attributed to genetic polymorphisms in drug transporters and transporter-mediated drug–drug interactions [14,15,16]. This highlights the need to investigate the mechanisms underlying metformin transport and its interactions with other drugs to optimize its application in periodontal tissue engineering.

Chemically, metformin is a hydrophilic small molecule base that predominantly exists as a cation (>99.9%) at physiological pH, which limits its ability to passively diffuse across cell membranes [17,18]. Instead, its absorption, hepatic uptake, and renal excretion rely heavily on organic cation transporters (OCTs), which play critical roles in the pharmacokinetics, energy metabolism, and drug–drug interactions of metformin [19,20]. Among the three OCT isoforms—OCT1, OCT2, and OCT3—these transporters are broadly expressed in the intestine, liver, kidneys, and other tissues [21,22,23], where they regulate metformin’s absorption, distribution, and elimination [24,25]. While studies on OCT1/2/3 transporters have extensively explored their tissue-specific expression and subcellular localization, their role in stem cells remains poorly understood.

Building on these findings, the current study aims to: (1) investigate the expression and functional activity of OCTs in hPDLSCs; (2) evaluate the role of OCT-mediated metformin transport in the osteogenic and cementogenic differentiation of hPDLSCs in vitro; and (3) assess the impact of cimetidine, an OCT inhibitor, on the regenerative efficacy of metformin in vivo. The following hypotheses were tested: (1) Functional OCT expression in hPDLSCs is critical for intracellular metformin uptake, enabling its osteogenic and cementogenic effects; (2) Cimetidine inhibits OCT-mediated metformin transport in hPDLSCs, reducing metformin accumulation and impairing its osteogenic and cementogenic potential in vitro; (3) In vivo, cimetidine attenuates metformin-enhanced periodontal tissue regeneration by interfering with its OCT-mediated transport.

## 2. Materials and Methods

### 2.1. Cell Sources and Treatment

hPDLSCs were obtained from healthy 18–22-year-old patients undergoing premolar extractions for orthodontic treatment, with no history of periodontal disease or caries. hPDLSCs obtained from six donors were named as hPDLSCs1–6. This study was approved by the Ethics Committee of Beijing Stomatological Hospital, Capital Medical University (No. CMUSH-IRB-KJ-PJ-2022-43), and written informed consent was obtained. Isolation followed established protocols [26] within 2 h post-extraction. Cells were subcultured at a 1:3 ratio, and passages 3–5 were used for the experiments. The growth medium included MEM-α (HyClone, Logan, UT, USA) with 10% FBS (Gibco, Thermo Fisher Scientific, Waltham, MA, USA) and 1% penicillin/streptomycin (Gibco). The mineralizing induction medium consisted of MEM-α, 10% FBS, 1% penicillin/streptomycin, 100 nM dexamethasone, 10 mM β-glycerophosphate, and 100 μM ascorbic acid (all from Sigma-Aldrich, St. Louis, MO, USA) [9].

### 2.2. Western Blot Analysis

Total protein was extracted using RIPA buffer with PMSF (100:1) (Beyotime, Shanghai, China), followed by centrifugation (4 °C, 14,000 rpm, 5 min) to collect the supernatant. Protein concentrations were normalized, mixed with 5× SDS-PAGE loading buffer (Beyotime), and denatured. Samples were loaded onto 4–12% pre-cast gels for SDS-PAGE and transferred to PVDF membranes using a semi-dry transfer system. Membranes were blocked with QuickBlock™ buffer (Beyotime), incubated overnight with the primary antibody at 4 °C, and washed with TBST. Secondary antibody incubation was performed for 1 h at room temperature, followed by TBST washes. Proteins were visualized using an ECL substrate [27].

### 2.3. Immunofluorescence Staining

Cells were fixed with 4% paraformaldehyde for 15 min and subsequently washed thrice with PBS. Permeabilization was achieved using 0.2% Triton X-100 in PBS for 15 min at room temperature, followed by blocking with 2% BSA (Solarbio, Beijing, China) for 30 min. Primary antibodies were applied overnight at 4 °C; rabbit anti-SLC22A1/OCT1, rabbit anti-SLC22A2/OCT2, and rabbit anti-SLC22A3/OCT3 (all from Bioss, Beijing, China). Following thorough washing, fluorescent secondary antibodies were introduced and incubated at 37 °C in the dark for 1 h, including FITC-conjugated goat anti-rabbit IgG (H + L) and Cy3-conjugated goat anti-rabbit IgG (H + L) (both from Beyotime). Nuclei were stained with DAPI (Beyotime) for 10 min at room temperature [28].

### 2.4. Cell Proliferation Assay

The CCK-8 assay was used to evaluate hPDLSC proliferation under different treatment conditions over 10 days. The experimental groups included the following: control (100 μM metformin), cimetidine (100 μM metformin + 100 μM cimetidine), and shOCT1 (hPDLSCs with lentivirus-mediated OCT1 knockdown, 100 μM metformin). The metformin concentration (100 μM) was selected based on our previous study, which demonstrated this dose to be optimal for promoting proliferation, migration, osteogenic, and cementogenic differentiation of hPDLSCs in vitro [29]. Cells (4 × 10^3^/100 μL) were seeded in 96-well plates (6 replicates per group). After replacing the medium, OD values at 450 nm were measured on days 1, 4, 7, and 10. CCK-8 solution (Beyotime) (10%) was added to each well and incubated at 37 °C for 60 min before measurement. Data were analyzed to plot proliferation curves [30].

### 2.5. Metformin Uptake Assay

hPDLSCs (2 × 10^5^ cells/well) were seeded in 6-well plates and cultured to 70–80% confluency. After pre-treatment with KRH buffer (37 °C, 30 min), cells were divided into three groups as follows: (1) Control: KRH buffer + 100 μM metformin; (2) Cimetidine group: KRH buffer + 100 μM metformin + 100 μM cimetidine; (3) shOCT1 group: KRH buffer + 100 μM metformin in hPDLSCs with OCT1 knockdown via lentivirus-mediated shRNA transduction. Cells were incubated with 2 mL of the corresponding solution at 37 °C. After incubation, cells were washed with ice-cold KRH buffer and lysed using an ultrasonic cell disruptor. The lysates were centrifuged at 14,000× *g* for 30 min in 3 kDa MWCO Amicon^®^ Ultra-0.5 ultrafiltration tubes (Merck Millipore, Burlington, MA, USA). The filtrates were collected and analyzed for metformin concentration using HPLC [31,32].

### 2.6. OCT1 Knockdown via Lentivirus-Mediated shRNA Transduction

Lentiviral particles carrying shRNA targeting OCT1 were prepared and transduced into hPDLSCs following the manufacturer’s protocol (GeneChem, Shanghai, China). hPDLSCs were seeded in 6-well plates (2 × 10^5^ cells per well) and transduced with lentiviral particles at a multiplicity of infection (MOI) of 10 in the presence of HitransG P (GeneChem) as the viral transduction enhancer. After 16 h, the medium was replaced with fresh growth medium, and cells were cultured for an additional 48 h. Cells were then subjected to puromycin (2 μg/mL) selection for 48 h to ensure stable transduction. Successful OCT1 knockdown was confirmed by Western blot analysis [33].

### 2.7. Quantitative Real Time-Polymerase Chain Reaction (qRT-PCR)

RNA was isolated from cells utilizing TRIzon Reagent (Cowin Biosciences, Beijing, China). Following this, cDNA synthesis was executed using PrimeScript^TM^ RT Master Mix (Takara Bio Inc., Shiga, Japan), as directed by the manufacturer. Quantitative RT-PCR assays were conducted with TB Green Premix Ex Taq II (Takara) on a CFX-96 Real-Time PCR Detection System (Bio-Rad Laboratories, Hercules, CA, USA). Expression levels of target genes were quantified relative to GAPDH (the reference gene), employing the standard 2^−ΔΔCt^ method [33]. Details of the primers employed can be found in Table 1.

### 2.8. ALP Activity Assay and Alizarin Red S (ARS) Staining

hPDLSCs (1 × 10^5^ cells/mL) were seeded in 6-well plates and cultured in an osteogenic medium. For ALP staining, cells were cultured for 7 days, fixed with 4% paraformaldehyde for 30 min, and stained overnight at room temperature with BCIP/NBT substrate. For ALP activity, cells were lysed after 7 days of culture, and the supernatant was mixed with detection buffer and p-nitrophenol substrate. OD values at 405 nm were measured using a microplate reader and were normalized to the protein concentration. For ARS staining, cells were cultured for 21 days, fixed, and stained with Alizarin Red S. Mineral deposition was observed microscopically, and quantification was performed by extracting ARS with 5% cetylpyridinium chloride and measuring absorbance at 562 nm [34,35].

### 2.9. In Vivo Periodontal Defect Model in Rats

Thirty 8-week-old male rats (200–250 g) were randomly divided into five groups (n = 6 per group). The experimental groups and treatment regimens are summarized in Table 2. The animal use protocol was approved by the Animal Ethical and Welfare Committee of Beijing Stomatological Hospital, Capital Medical University (KQYY-202211-009). Under general anesthesia, standardized box-shaped periodontal defects (5 × 3 × 1.5 mm) were created 1 mm below the alveolar crest of the first molars. The procedure involved the removal of buccal alveolar bone, periodontal ligament, and root surfaces. Cimetidine was administered to rats in Groups 4 and 5 via drinking water at a dose of 100 mg/kg/day [36]. From the acclimation period to the end of the experiment, water consumption and body weight were monitored twice weekly to calculate the appropriate cimetidine concentration in drinking water. After six weeks, the animals were sacrificed, and periodontal tissue regeneration at the defect sites was evaluated [37].

### 2.10. Micro-CT and Histomorphometric Analyses

Rat mandibles were fixed in 4% paraformaldehyde and scanned using a SkyScan 1276 Micro-CT system (Bruker, Kontich, Belgium) with a source voltage of 85 kV, a current of 160 μA, an exposure time of 450 ms, and a resolution of 9 μm. Three-dimensional reconstructions were generated using CTvox (Bruker), and sectional views were analyzed with DataViewer (Bruker). Following Micro-CT, samples were decalcified, embedded in paraffin, sectioned at 5 μm, and stained with HE. New alveolar bone, defined as bone islands within the defect, was quantified as a percentage of the defect area using ImageJ (version 1.54g, National Institutes of Health, Bethesda, MD, USA). New cementum, characterized by mineralized tissue with embedded collagen fibers on the root surface, was quantified as the percentage of the corresponding dentin area in the section [37,38].

### 2.11. Data Analysis

Statistical analyses were conducted using GraphPad Prism software (version 8.0.1, GraphPad Software, San Diego, CA, USA). Prior to the application of parametric tests, the normality of data distribution was assessed using the Shapiro–Wilk test. Group comparisons were performed using one-way analysis of variance (ANOVA) followed by Tukey’s post hoc test. For time-dependent measurements, two-way ANOVA was used to assess interactions between variables. A *p*-value of <0.05 was considered statistically significant. In the figures, different lowercase letters (e.g., a, b, c) indicate statistically significant differences between groups; groups not sharing the same letter are significantly different (*p* < 0.05).

## 3. Results

### 3.1. Differential Expression and Functional Activity of OCTs in hPDLSCs

**Figure 1:**
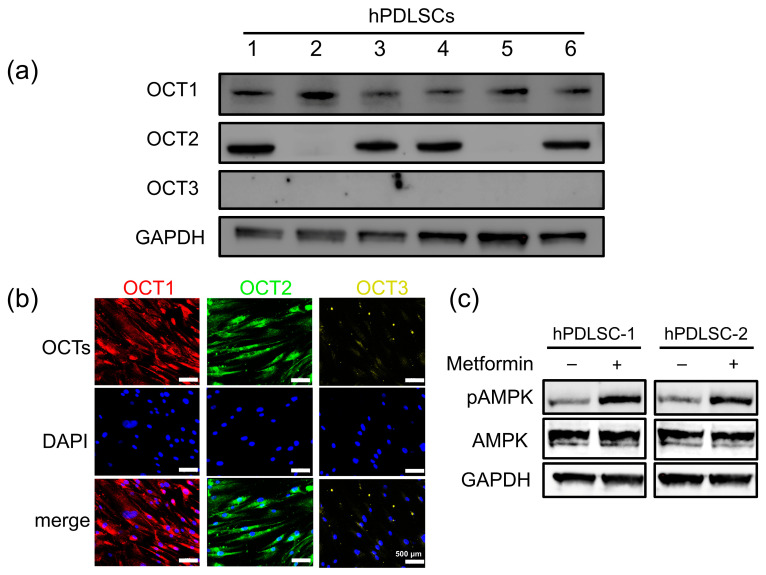
Differential expression and functional activity of OCTs in hPDLSCs. (**a**) Western blot analysis of hPDLSCs from six human donors revealed differential expression of OCT isoforms. GAPDH served as the loading control. (**b**) Immunofluorescence staining confirmed OCT1 and OCT2 localization in hPDLSCs, with no detectable OCT3 expression. Scale bar: 500 μm. (**c**) Western blot analysis of AMPK phosphorylation (pAMPK) in hPDLSC-1 (OCT1/2-expressing) and hPDLSC-2 (OCT1-only) following metformin treatment. GAPDH was used as the loading control. (original Western Blot images see supplementary Figure S2)

The isolated cells were identified as hPDLSCs by assessing their morphology, surface antigen expression, and tri-lineage differentiation potential (Figure S1). Western blot analysis of hPDLSCs from six human donors revealed that OCT1 was consistently expressed in all samples, while OCT2 was absent in hPDLSC-2 and hPDLSC-5, and OCT3 was not detected in any samples (Figure 1a). Immunofluorescence confirmed these findings, showing OCT1 and OCT2 localization in hPDLSC-1, with no detectable OCT3 expression (Figure 1b). To assess OCT functionality, metformin was added to hPDLSC-1 and hPDLSC-2 cultures, and AMPK phosphorylation (pAMPK) indicated metformin intracellular uptake. Western blot results showed increased pAMPK levels in both cell types after metformin treatment, indicating effective metformin transport despite differing OCT expression patterns (Figure 1c).

### 3.2. Role of OCTs in Metformin Uptake in hPDLSCs

**Figure 2:**
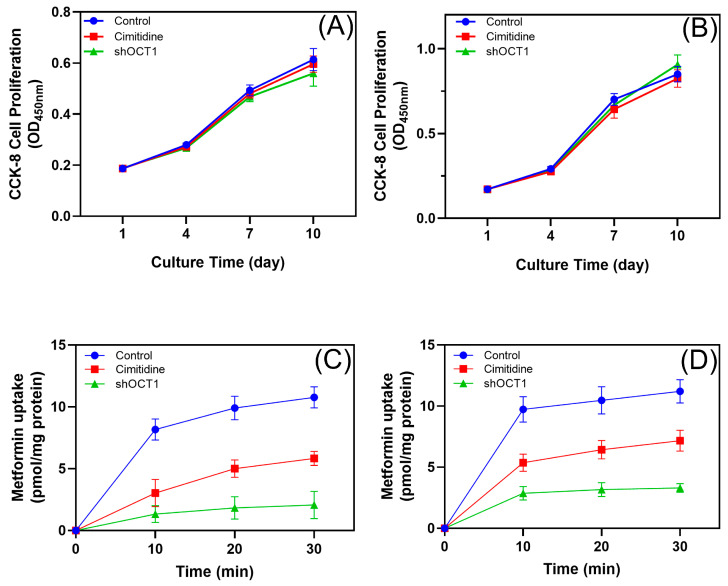
Effect of OCTs on metformin uptake. (**A**,**B**) Proliferation of hPDLSC-1 ((**A**), OCT1/2) and hPDLSC-2 ((**B**), OCT1-only) under three conditions: control (100 μM metformin), cimetidine (100 μM metformin + 100 μM cimetidine), and shOCT1 (hPDLSCs with OCT1 knockdown via lentivirus-mediated shRNA transduction, 100 μM metformin). No significant differences in proliferation were observed. (**C**,**D**) Metformin uptake in hPDLSC-1 (**C**) and hPDLSC-2 (**D**) assessed over 30 min. Data are presented as mean ± SD (n = 3).

CCK-8 assays were conducted in hPDLSC-1 (OCT1/2-expressing) (Figure 2A) and hPDLSC-2 (OCT1-only) (Figure 2B) over 10 days. No significant differences in cell proliferation were observed among the control, cimetidine-treated, and shOCT1 groups, indicating that OCT inhibition or knockdown does not affect hPDLSC growth. In hPDLSC-1, cimetidine reduced metformin uptake to 50–60% of the control (*p* < 0.01), while shOCT1 further decreased uptake to 20–30% of the control (*p* < 0.01) (Figure 2C). In hPDLSC-2, which expresses only OCT1, cimetidine and shOCT1 similarly impaired metformin uptake (Figure 2D). These results confirm that OCT1 is the primary transporter mediating metformin uptake in hPDLSCs despite different expression patterns.

### 3.3. Role of OCT1 in Metformin-Induced AMPK/ACC Activation and Osteogenic/Cementogenic Differentiation

**Figure 3:**
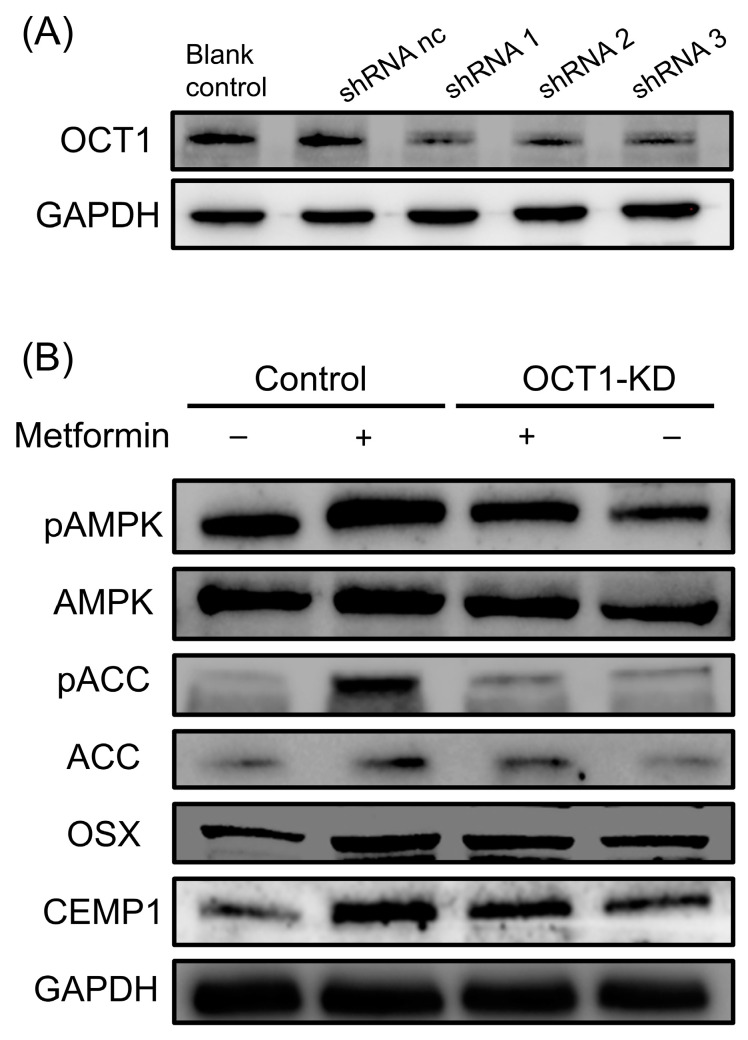
Role of OCT1 in metformin-induced AMPK/ACC activation and differentiation in hPDLSCs. (**A**) OCT1 knockdown in hPDLSCs was achieved via lentivirus-mediated shRNA transduction. GAPDH was used as the loading control. (**B**) Western blot analysis showing the effect of OCT1 knockdown on AMPK/ACC signaling and differentiation markers in hPDLSCs. Metformin treatment significantly increased levels of pAMPK and pACC in control hPDLSCs, along with the expression of OSX and CEMP1, while these effects were markedly reduced in OCT1-knockdown (OCT1-KD) cells. (original Western Blot images see supplementary Figure S3).

OCT1 was efficiently knocked down in hPDLSCs using lentivirus-mediated shRNA transduction, as confirmed by Western blot analysis (Figure 3A). In OCT1-knockdown (OCT1-KD) cells, metformin-induced AMPK phosphorylation (pAMPK) was significantly reduced compared to control cells, indicating that OCT1 is essential for metformin transport into hPDLSCs and subsequent AMPK activation (Figure 3B). Metformin treatment significantly increased the phosphorylation of ACC (pACC), a downstream effector of AMPK, while this activation was markedly suppressed in OCT1-KD cells (Figure 3B). Furthermore, metformin treatment upregulated the expression of osteogenic transcription factor *OSX* and cementogenic marker *CEMP1*, whereas this effect was attenuated in OCT1-KD cells (Figure 3B). These findings indicate that the AMPK/ACC signaling pathway, activated by metformin via OCT1, may regulate osteogenic and cementogenic differentiation of hPDLSCs.

### 3.4. Cimetidine Inhibits Metformin-Induced Osteogenic and Cementogenic Differentiation

**Figure 4:**
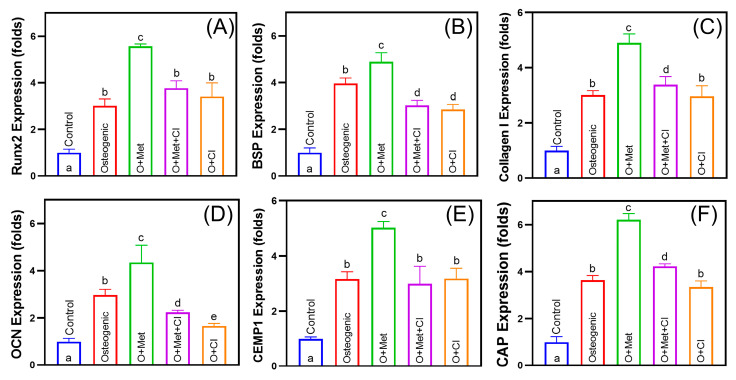
Effects of metformin and cimetidine on osteogenic and cementogenic marker expression in hPDLSCs. Quantitative RT-PCR analysis of osteogenic markers (*Runx2*, *BSP*, *Collagen I*, and *OCN*) and cementogenic markers (*CEMP1* and *CAP*) in hPDLSCs cultured under different conditions: growth medium (Control), osteogenic medium (Osteogenic), osteogenic medium with 100 μM metformin (O + Met), osteogenic medium with 100 μM metformin and 100 μM cimetidine (O + Met + CI), and osteogenic medium with 100 μM cimetidine alone (O + CI). (**A**) *Runx2* expression was significantly increased in the O + Met group compared to the osteogenic group, while cimetidine (O + Met + CI) partially reversed this effect. (**B**) *BSP* expression reached the highest level in the O + Met group; cimetidine reduced its expression to a level even lower than that observed in the osteogenic group. (**C**) *Collagen I* expression was markedly upregulated by metformin and suppressed by cimetidine to a level comparable to the osteogenic group. (**D**) *OCN* expression was strongly induced by metformin and significantly decreased by cimetidine treatment, resulting in levels even lower than those in the osteogenic group. (**E**) *CEMP1* expression was elevated in the O + Met group, whereas cimetidine attenuated this effect, bringing the expression close to that of the osteogenic group. (**F**) *CAP* expression peaked in the O + Met group and was reduced by cimetidine, although it remained higher than in the osteogenic group. Data are presented as mean ± SD. Different letters (such as a, b, and c) above the bars indicate statistically significant differences between groups (n = 5, *p* < 0.05). Bars sharing the same letter are not significantly different from each other.

Metformin (O + Met) significantly enhanced osteogenic marker expression, including *Runx2*, *BSP*, *Collagen I*, and *OCN*, by 1.2–1.8-fold compared to the osteogenic group (Figure 4A–D). Among these, *Runx2* showed the most prominent upregulation (1.8-fold). Cimetidine (O + Met + CI) partially reversed these effects, reducing the expression of *Runx2*, *BSP*, and *Collagen I* by approximately 31–38%, and decreasing *OCN* expression by 48%, resulting in levels even lower than the osteogenic group. For cementogenic markers, metformin significantly increased *CEMP1* and *CAP* expression by 1.6–1.7-fold (Figure 4E,F). Cimetidine treatment suppressed this upregulation, reducing *CEMP1* expression by 40% to a level similar to that of the osteogenic group, and decreasing *CAP* expression by 32%, though *CAP* remained slightly higher than osteogenic control (*p* < 0.05).

**Figure 5:**
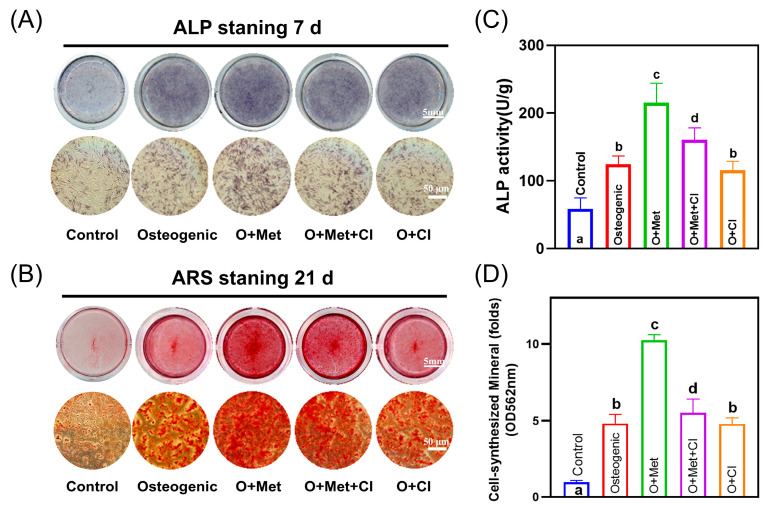
Effects of cimetidine on metformin-induced osteogenic differentiation of hPDLSCs. (**A**) Representative images of ALP staining in hPDLSCs after 7 days of mineralization induction under different conditions. (**B**) Representative images of Alizarin Red S (ARS) staining after 21 days to assess mineralized matrix formation. (**C**) Quantitative analysis of ALP activity after 7 days of treatment. (**D**) Quantitative measurement of cell-synthesized mineral content at 21 days by ARS staining. Data are presented as mean ± SD. Different letters (such as a, b, and c) above the bars indicate statistically significant differences between groups (n = 4, *p* < 0.05). Bars sharing the same letter are not significantly different from each other.

Accordingly, metformin (O + Met) increased ALP activity by 1.7-fold and mineralized matrix deposition by 2.1-fold compared to the osteogenic group. Cimetidine (O + Met + CI) reduced ALP activity and mineralization by 25% and 46% relative to O + Met (Figure 5). These findings confirm that metformin significantly promotes both osteogenic and cementogenic differentiation in hPDLSCs, while cimetidine attenuates these effects by inhibiting OCT-mediated metformin transport. This underscores the critical role of OCTs in facilitating metformin’s regenerative potential.

### 3.5. Cimetidine Attenuates Metformin-Induced Periodontal Regeneration In Vivo

**Figure 6:**
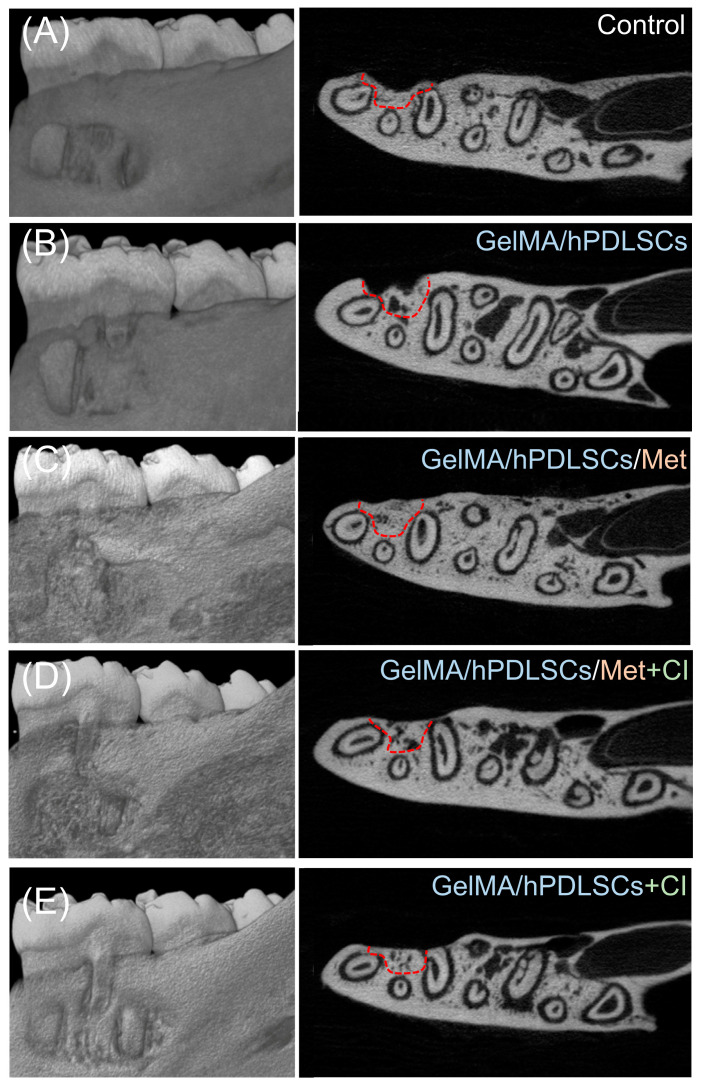
Micro-CT analysis of periodontal defect repair in rat models. Representative three-dimensional reconstruction (**left**) and cross-sectional images (**right**) of periodontal defects treated with (**A**) saline (control), (**B**) GelMA/hPDLSCs, (**C**) GelMA/hPDLSCs with metformin (GelMA/hPDLSCs/Met), (**D**) GelMA/hPDLSCs/Met and orally administered cimetidine (GelMA/hPDLSCs/Met + CI), and (**E**) GelMA/hPDLSCs with orally administered cimetidine (GelMA/hPDLSCs + CI). Dashed red lines outline the defect areas with periodontal regeneration.

**Figure 7:**
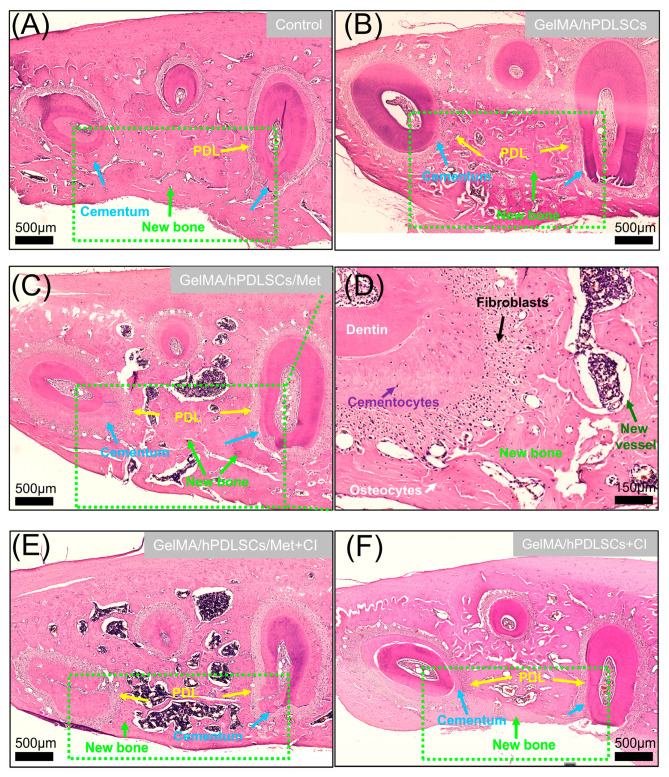
Histological analysis of periodontal regeneration in rat defects. (**A**–**C**,**E**,**F**) Representative HE-stained sections of periodontal defect sites in different treatment groups: (**A**) Control, showing limited new tissue formation; (**B**) GelMA/hPDLSCs, demonstrating moderate bone and cementum regeneration; (**C**) GelMA/hPDLSCs/Met, showing enhanced regeneration with significant new bone and cementum formation; (**D**) Higher magnification of the mesial root of first molar in (**C**), showing a well-organized periodontal structure in the GelMA/hPDLSCs/Met group. Cementocytes embedded in new cementum (purple arrow), osteocytes in newly formed bone (white arrow), and new blood vessels surrounding the new bone (green arrow) indicate active tissue regeneration. Scale bar: 150 μm. (**E**) GelMA/hPDLSCs/Met + CI and (**F**) GelMA/hPDLSCs + CI, both exhibiting reduced regeneration due to cimetidine treatment. Scale bars: 500 μm.

**Figure 8:**
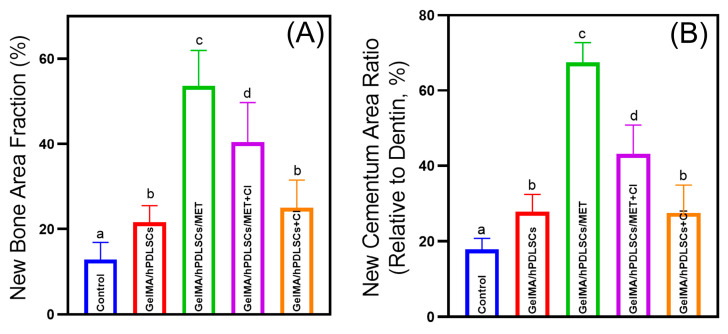
Quantitative analysis of periodontal regeneration in rat defects. (**A**) New bone area fraction (%) in different treatment groups: control, GelMA/hPDLSCs, GelMA/hPDLSCs/Met, GelMA/hPDLSCs/Met + CI, and GelMA/hPDLSCs + CI. The GelMA/hPDLSCs/Met group showed the highest new bone formation, while cimetidine treatment (GelMA/hPDLSCs/Met + CI) significantly reduced bone regeneration. (**B**) New cementum area ratio (relative to dentin, %). Consistent with new bone formation, the GelMA/hPDLSCs/Met group exhibited the most substantial new cementum formation, with cimetidine treatment attenuating this effect. Data are presented as mean ± SD. Different letters (such as a, b, and c) above the bars indicate statistically significant differences between groups (n = 5, *p* < 0.05). Bars sharing the same letter are not significantly different from each other.

The results of Micro-CT analysis are shown in Figure 6. Both 3D reconstructions and cross-sectional views revealed that the GelMA/hPDLSCs/Met group exhibited the most significant periodontal defect repair. However, the addition of cimetidine (GelMA/hPDLSCs/Met + CI), administered via drinking water at 100 mg/kg/day, substantially reduced the regenerative outcomes, as reflected by smaller new bone and cementum areas. Histological analysis (Figure 7) confirmed these observations. Quantitatively, the new bone area fraction in the GelMA/hPDLSCs/Met group was 53.6 ± 7.4%, significantly higher than in the GelMA/hPDLSCs group (21.6 ± 3.4%). Cimetidine reduced new bone formation to 40.4 ± 8.3% in the GelMA/hPDLSCs/Met + CI group (n = 5, *p* < 0.05) (Figure 8A). Similarly, the new cementum area ratio in the GelMA/hPDLSCs/Met group was 67.5 ± 4.7%, while cimetidine decreased it to 43.1 ± 6.9% (n = 5, *p* < 0.05) (Figure 8B). These results demonstrate that metformin significantly enhances both osteogenesis and cementogenesis in vivo. Cimetidine attenuates these regenerative effects, highlighting the critical role of OCTs in the therapeutic effects of metformin on periodontal tissue repair.

## 4. Discussion

In this study, we demonstrated for the first time that functional OCTs are differentially expressed in hPDLSCs and play a crucial role in metformin uptake, mediating its effects on promoting periodontal regeneration. The following hypotheses were validated: (1) Functional OCT expression in hPDLSCs is essential for intracellular metformin uptake, enabling its osteogenic and cementogenic effects; (2) Cimetidine inhibits OCT-mediated metformin transport in hPDLSCs, reducing intracellular metformin accumulation and impairing its osteogenic and cementogenic potential in vitro; (3) In vivo, cimetidine attenuates metformin-enhanced periodontal tissue regeneration by interfering with its OCT-mediated transport. This study highlights the importance of OCTs in metformin-based periodontal therapies, emphasizing the need for precision medicine strategies.

Metformin, beyond its glucose-lowering effects, has shown promise in promoting periodontal regeneration. Pradeep et al. [5] demonstrated that local application of metformin gel reduced attachment loss and probing depth after periodontal therapy. Bak et al. [39] reported significantly less alveolar bone loss in ligature-induced periodontitis in metformin-treated rats. Ren et al. [40] further highlighted the potential of metformin carbon dots in enhancing periodontal bone regeneration via ERK/AMPK pathway activation, showing metformin’s regenerative potential in periodontal therapy. In our study, locally applied metformin-loaded GelMA hydrogel effectively promoted periodontal tissue regeneration in periodontal defects of rats.

Previous studies have demonstrated that OCT transporter expression is both species- and tissue-specific, closely linked to their functional roles [41]. OCT1 and OCT2 exhibit relatively restricted expression patterns, limited to specific organs or tissues, while OCT3 is more broadly expressed across various tissues [21,22,23]. In our study, OCT1 and OCT2 were detected in hPDLSCs. Similarly, Al Jofi et al. [42] reported differential expression of OCT1, OCT2, and OCT3 in human umbilical cord mesenchymal stromal cells (hUC-MSCs) derived from different parental sources. Wang et al. [27] further showed that iPSC-MSCs exclusively express OCT1. These findings highlight the variability in OCT expression patterns among stem cells of different parental and tissue origins.

Accumulating evidence highlights the critical role of OCTs in mediating the cellular transport of metformin. Wang et al. [27] reported that co-incubation of cells with 10 μM cimetidine for 10 min reduced metformin uptake to 60–70% of the control level. Similarly, Al Jofi et al. [42] demonstrated that treatment with the pan-OCT inhibitor quinidine significantly impaired metformin uptake in hUC-MSCs. In osteoblasts, Ma et al. [43] showed that phenformin and cimetidine markedly inhibited metformin uptake, decreasing it from 744 ± 17 pmol/mg protein/10 min to approximately 134–146 pmol/mg protein/10 min. Consistent with these findings, our study demonstrated that cimetidine reduced metformin accumulation in hPDLSCs to 50–60% of control levels, and OCT1 knockdown further decreased uptake to 20–30%, confirming the essential role of OCTs in regulating metformin transport in periodontal ligament stem cells.

In the present study, we found that OCTs facilitate metformin-mediated osteogenesis and cementogenesis in hPDLSCs. Previous research also confirmed that the functional state of OCT transporters critically influences the therapeutic effects of metformin. Shu et al.’s study [44] found that deletion of OCT1 resulted in a reduction in the effects of metformin on AMPK phosphorylation and gluconeogenesis in mouse hepatocytes. Genetic variation in the OCT2 plays an important role in the renal elimination of metformin [45]. Additionally, Di Pietro et al. [28] demonstrated that metformin’s regulation of AMPK phosphorylation and VEGF expression in rat granulosa cells relies on OCT activity, as transporter inhibition reversed these effects. These findings highlight the essential role of functional OCTs in modulating metformin’s therapeutic efficacy.

The metabolic regulator AMPK has emerged as a central mechanism underlying the diverse effects of metformin [46,47]. Through the phosphorylation of acetyl-CoA carboxylase 1 (ACC1) and ACC2, AMPK regulates fatty acid synthesis and cellular energy metabolism [48]. Cellular energy metabolism plays a pivotal role in bone metabolism and repair. In primary osteoblasts, the AMPK/ACC axis was involved in the protective effects of resveratrol on dexamethasone-induced osteoblast damage [49]. A previous study reported that isomangiferin promotes the AMPK/ACC pathway of rat bone marrow mesenchymal stem cells and therefore contributes to bone healing [50]. Our study also suggests that the AMPK/ACC signaling pathway may contribute to the regulation of osteogenic and cementogenic differentiation in hPDLSCs.

Metformin is known to interact with a wide range of drugs, with 333 documented drug–drug interactions (DDIs), including 13 classified as major and 293 as moderate [51]. Among these, cimetidine is categorized as a major interaction, often necessitating adjustments to co-administration regimens [15,32]. Cimetidine, an H2 receptor antagonist commonly used to treat gastrointestinal disorders, has been identified as an OCT inhibitor [25,32]. Cimetidine was reported to increase the plasma metformin concentration–time area under the curve (AUC) by approximately 50% and decreased its 24-h renal clearance by 27% [52]. These studies raise concerns about the impact of OCT-mediated drug–drug interactions on metformin’s therapeutic efficacy [15,53].

Drug–drug interactions between cimetidine and metformin have been previously reported to influence the therapeutic effects of metformin. Ailabouni et al. [54] conducted a clinical pharmacokinetic study in sixteen healthy adults, where participants were administered metformin (50 mg) alone and in combination with cimetidine (400 mg). Co-administration resulted in a 24% increase in systemic plasma exposure to metformin and an 8% reduction in renal clearance, indicating that OCT inhibition alters metformin pharmacokinetics. Rieko et al. [55] reported that metformin is acutely transported into skeletal muscle cells via OCTs, where it activates AMPKα1 and α2 in both fast- and slow-twitch muscle fibers. Importantly, cimetidine suppressed metformin-induced AMPK phosphorylation and 3-O-methyl-D-glucose (3MG) uptake, further confirming the functional consequences of OCT inhibition.

In addition, José et al. [56] analyzed OCT1 and OCT2 gene expression in 118 human adipose tissue samples and found that increased OCT1 expression in obese individuals may enhance metformin action. Blocking OCT-mediated transport with cimetidine reversed these effects. Consistent with these observations, our study demonstrated that in the group co-treated with metformin and cimetidine, the osteogenic and cementogenic effects of metformin on hPDLSCs were significantly attenuated. These findings suggest that in patients concurrently receiving cimetidine or similar OCT inhibitors, the regenerative potential of metformin-loaded periodontal tissue engineering scaffolds may be substantially compromised.

While OCT-mediated metformin uptake regulates osteogenic and cementogenic differentiation of hPDLSCs, it should be noted that periodontal regeneration in vivo involves not only transplanted cells but also a substantial population of host-derived cells. Following scaffold implantation, endogenous stem or progenitor cells from the surrounding periodontal ligament, alveolar bone, and bone marrow are recruited to the defect site and actively participate in tissue regeneration [8,10]. Therefore, investigating the expression and functionality of OCTs in these host cell populations is equally important. Moreover, beyond promoting osteogenesis and cementogenesis, metformin has been shown to regulate a variety of cellular functions, such as enhancing cell proliferation and migration, modulating inflammatory responses, and promoting angiogenesis [3,6]. Whether these additional biological effects of metformin are similarly dependent on OCT-mediated transport remains an open question and warrants further investigation.

Our findings suggest the clinical importance of OCTs in metformin-based periodontal therapies, particularly in the context of precision and personalized medicine. The expression of functional OCTs in hPDLSCs highlights their potential as optimal seed cells when combining metformin as a bioactive factor in periodontal regeneration. However, individual variability in OCT expression or the use of OCT-inhibiting medications, such as cimetidine, could significantly affect treatment outcomes. These results suggest that integrating precision medicine principles, including personalized assessments of OCT activity, gene-editing-based tissue engineering, and the avoidance of adverse drug interactions, could enhance the efficacy and predictability of metformin-based periodontal regeneration therapies.

## 5. Conclusions

This study demonstrates, for the first time, that functional OCT transporters in hPDLSCs are essential for mediating metformin uptake and its osteogenic and cementogenic effects. OCT1 plays a key role, with its knockdown reducing metformin uptake to 20–30% of control levels. Metformin activates the AMPK/ACC signaling pathway, which influences the osteogenic and cementogenic differentiation of hPDLSCs. Cimetidine inhibited metformin-induced osteogenesis by 31–48% and cementogenesis by 32–40% in vitro, while in vivo, it reduced alveolar bone and cementum formation by 25% and 36%, respectively. These findings emphasize the importance of OCTs in metformin-based periodontal therapies, providing valuable insights for seed cell selection, drug design, and minimizing adverse drug interactions.

## Figures and Tables

**Table 1:** List of primer sequences used in qRT-PCR.

Gene	Forward Primer (5′ to 3′)	Reverse Primer (5′ to 3′)
*OCN*	GCAAAGGTGCAGCCTTTGTG	GGCTCCCAGCCATTGATACAG
*RUNX2*	TCTGGCCTTCCACTCTCAGT	GACTGGCGGGGTGTAAGTAA
*Collagen I*	CTGACCTTCCTGCGCCTGATGTCC	GTCTGGGGCACCAACGTCCAAGGG
*BSP*	GAACCACTTCCCCACCTTTT	TCTGACCATCATAGCCATCG
*CAP*	CCTGGCTCACCTTCTACGAC	CCTCAAGCAAGGCAAATGTC
*CEMP1*	GGGCACATCAAGCACTGACAG	CCCTTAGGAAGTGGCTGTCCAG
*GAPDH*	GCACCGTCAAGGCTGAGAAC	ATGGTGGTGAAGACGCCAGT

**Table 2:** Experimental groups and corresponding treatments in the rat periodontal defect model.

Group	Treatment Description	Cimetidine Administration
(1) Control	0.9% NaCl	no
(2) GelMA/hPDLSCs	GelMA hydrogel with hPDLSCs (5 × 10^6^/mL)	no
(3) GelMA/hPDLSCs/Met	GelMA hydrogel with hPDLSCs (5 × 10^6^/mL) and metformin (100 μM)	no
(4) GelMA/hPDLSCs/Met + CI	GelMA hydrogel with hPDLSCs (5 × 10^6^/mL) and metformin (100 μM)	100 mg/kg/day in water
(5) GelMA/hPDLSCs + CI	GelMA hydrogel with hPDLSCs (5 × 10^6^/mL)	100 mg/kg/day in water

## Data Availability

The original contributions presented in this study are included in the article/Supplementary Materials. Further inquiries can be directed to the corresponding authors.

## Supplementary Materials

The following supporting information can be downloaded at: https://www.mdpi.com/article/10.3390/biom15050663/s1, Figure S1: Cell culture and identification of hPDLSCs; Figure S2: Original western blot images related to Figure 1; Figure S3: Original western blot images related to Figure 3.

## Abbreviations

The following abbreviations are used in this manuscript:

OCTs	Organic cation transporters
hPDLSCs	Human periodontal ligament stem cells
HPLC	High-performance liquid chromatography
PDLSCs	Periodontal ligament stem cells
ARS	Alizarin Red S
hUC-MSCs	Human umbilical cord mesenchymal stromal cells
ACC1	Acetyl-CoA carboxylase 1
DDIs	Drug–drug interactions
AUC	Area under the curve
