# Supplementary material for: Combination of Periodontal Ligament Stem Cells and Metformin via Organic Cation Transporters for Periodontal Regeneration in Rats
Source: Biomolecules. 2025 May 3;15(5):663. doi: 10.3390/biom15050663 (PMC12108652; doi:10.3390/biom15050663)
Supplement: Supplementary file 1 [file biomolecules-15-00663-s001.zip › biomolecules-3556987-supplementary.pdf]

SUPPLEMENTARY FIGURE TITLES AND LEGENDS for

Qiao et. al., Biomolecules

" Combination of periodontal ligament stem cells and metformin via organic cation transporters for periodontal regeneration in rats. "

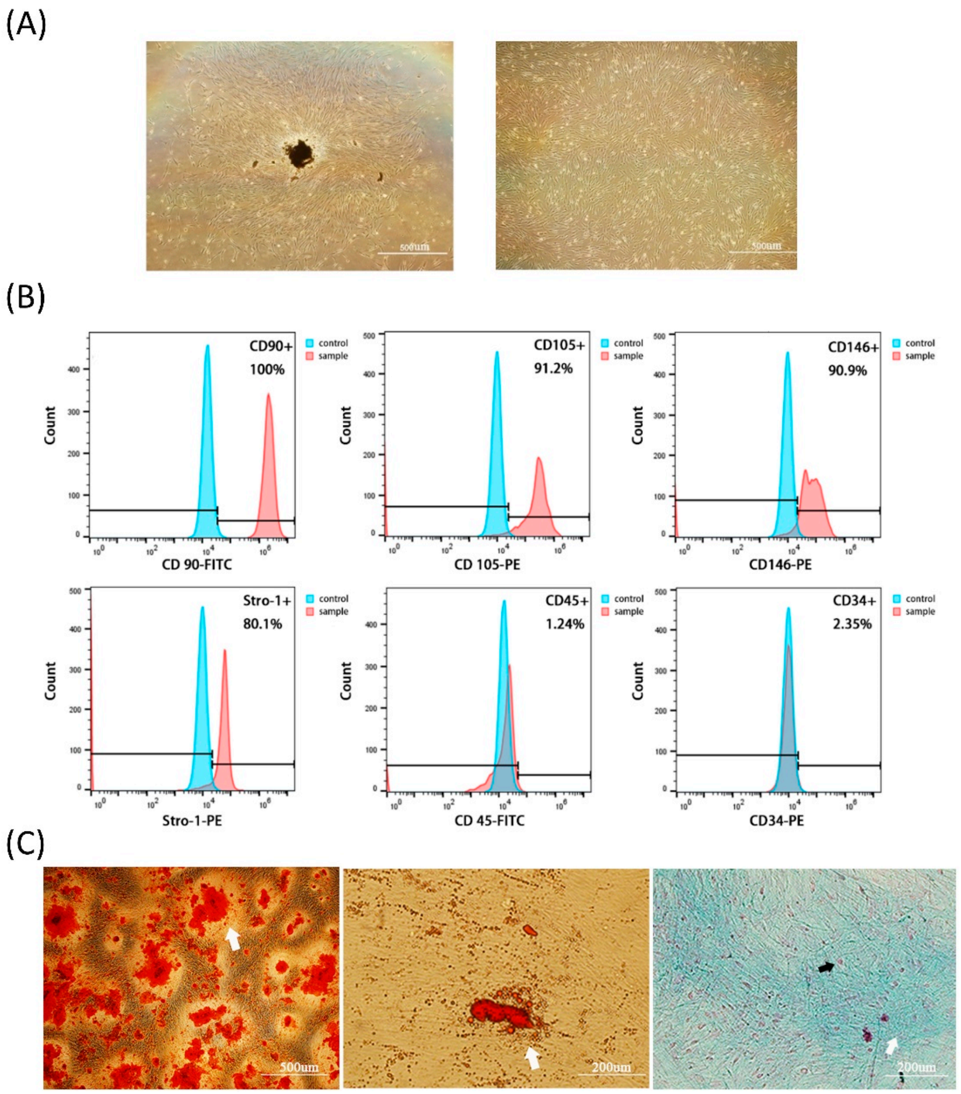

Figure S1. Cell culture and identification of hPDLSCs. (A) The morphology of primary and passaged hPDLSCs under a microscope. (B) Flow cytometry analysis of isolated cell surface antigen. Blue curves represent control samples, while red curves represent the test samples. (C)Tri-lineage differentiation potential of isolated stem cells. From left to right: osteogenic differentiation stained with Alizarin Red showing

mineralized nodules (indicated by arrows); adipogenic differentiation stained with Oil Red O showing lipid droplets (indicated by arrows); and chondrogenic differentiation stained with Alcian Blue showing proteoglycan-rich areas (indicated by arrows).

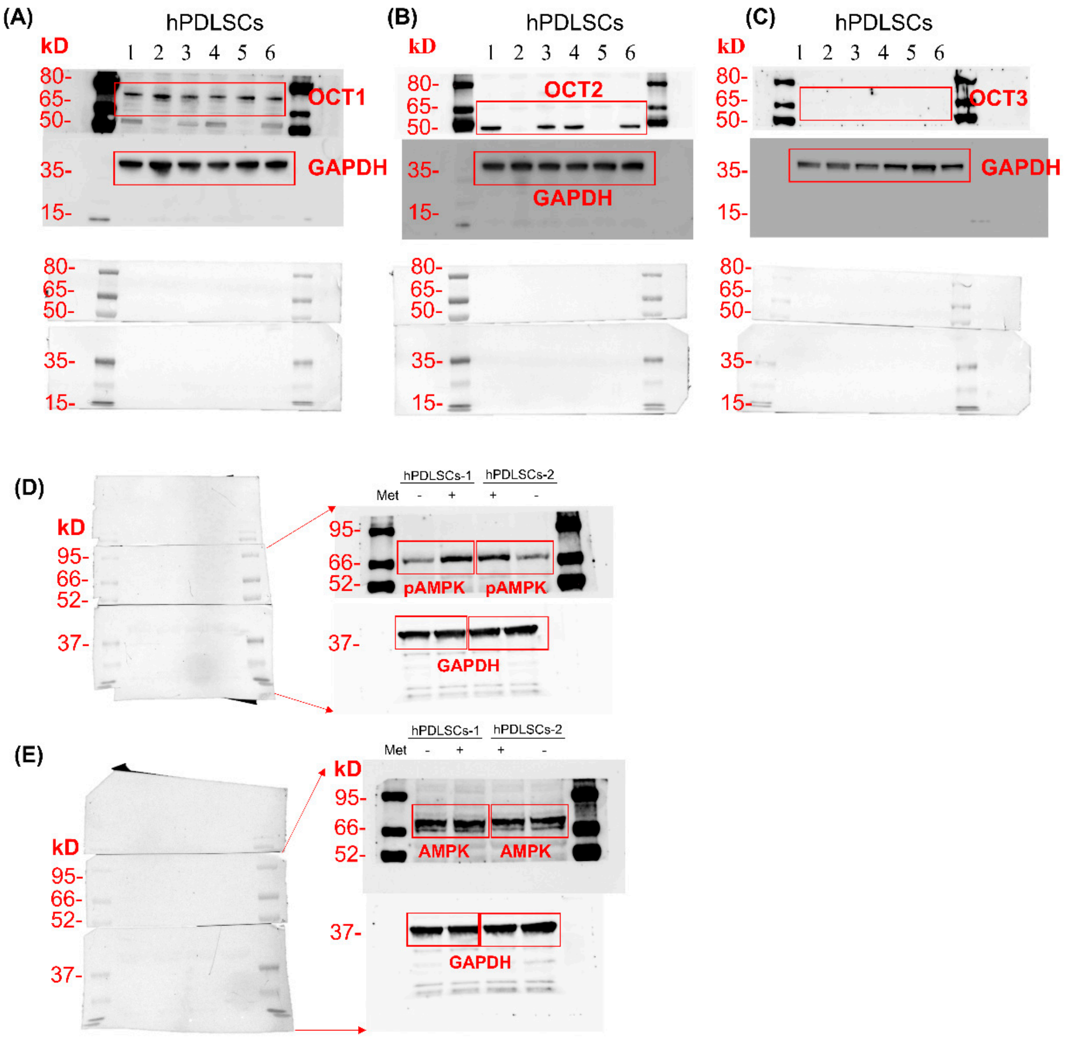

Figure S2. Original western blot images related to Figure 1. (A-C) Original western blot images related to Figure 1A. (D, E) Original western blot images related to Figure 1C.

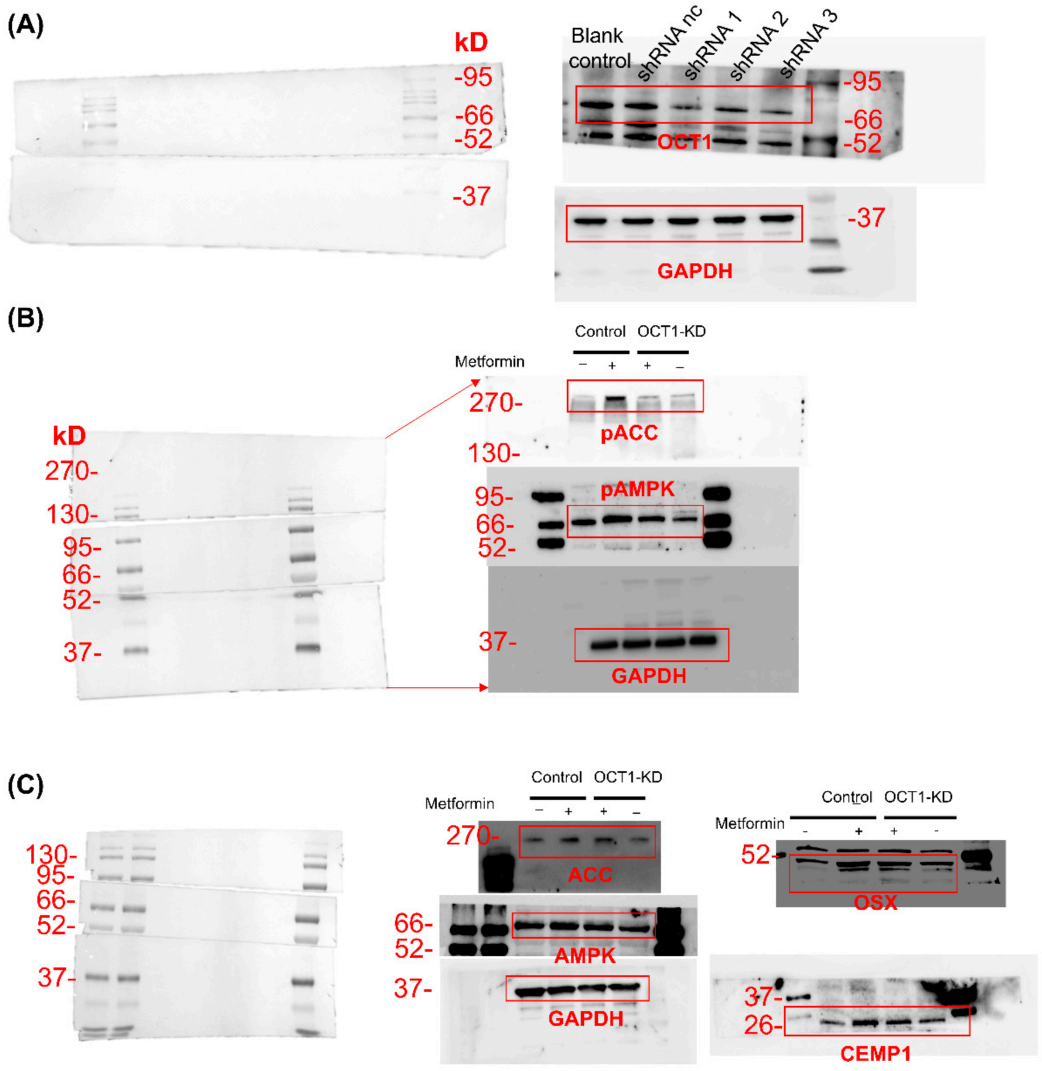

Figure S3. Original western blot images related to Figure 3. (A) Original western blot images related to Figure 3A. (B, C) Original western blot images related to Figure 3B.
